# Factors influencing root resorption in retained mandibular second deciduous molars with congenital absence of second premolars: a cross-sectional study

**DOI:** 10.1186/s40510-024-00512-8

**Published:** 2024-04-01

**Authors:** Keita Ishizuka, Chiho Kato, Akiyo Fujita, Eri Misawa-Omori, Takashi Ono

**Affiliations:** Department of Orthodontic Science, Graduate School of Medical and Dental Sciences, Tokyo Medical and Dental University (TMDU), 1-5-45 Yushima Bunkyo-ku, Tokyo, 113-8510 Japan

**Keywords:** Retained deciduous tooth, Congenital absence, Root resorption, Bite force

## Abstract

**Background:**

There are currently no studies that quantitatively compare the relationship of root resorption to the patient’s systemic history or craniofacial and intraoral morphology, especially in relation to possible host factors. Thus, this study aimed to clarify the factors associated with root resorption in retained mandibular second deciduous molars with the congenital absence of second premolars and predict the prognosis of retained mandibular second deciduous molars.

**Methods:**

A cohort of 5547 patients who visited the orthodontic clinic at Tokyo Medical and Dental University Dental Hospital between 2013 and 2022 was screened. Lateral cephalometric radiographs, panoramic radiographs, upper and lower dental models, and orthodontic treatment questionnaires were used as reference materials to apply the inclusion and exclusion criteria. Ultimately, 111 patients were included in the analyses. The patients were divided into two groups based on the root resorption levels of the retained mandibular second deciduous molars. Those with less root resorption were classified under the good condition (GC) group, whereas those with more root resorption were classified under the poor condition (PC) group. Demographic, clinical, and cephalometric parameters were compared between the groups. A multivariate logistic regression model was used to predict the probability of root resorption.

**Results:**

The prevalence of congenitally missing mandibular second premolars with persistent mandibular second deciduous molars was 2.0%. In a total of 111 patients, eighty-three teeth (53.2%) were classified into the GC group, whereas 73 teeth (46.8%) were classified into the PC group. The Frankfort-mandibular plane angle (FMA) [odds ratio (OR): 0.87], Frankfort-mandibular incisor angle (FMIA) (OR: 0.93), overbite (OR: 1.38), adjacent interdental space (OR: 1.46), distance from occlusal plane (OR: 0.80), and caries treatment (OR: 7.05) were significantly associated with the root resorption of the retained mandibular second deciduous molars.

**Conclusions:**

Our findings suggest that skeletal morphology, oral morphological patterns, and history contribute to root resorption in retained mandibular second deciduous teeth with congenital absence of subsequent permanent teeth.

## Background

Retained deciduous teeth are those that are retained in place past the normal replacement period without the eruption of the permanent teeth. Moreover, 90% of these patients have congenital absence of succeeding permanent teeth [1]. In Asia, the prevalence of the congenital absence of permanent teeth is estimated at 6.3% [2]. Except for the third molars, the mandibular second premolars have the highest incidence of congenital absence, with 25.9% of patients with hypodontia and 88.2% of patients with oligodontia having congenitally absent mandibular second premolars. Therefore, retention of the preceding deciduous tooth, mandibular second deciduous molars, is frequently observed [3, 4].

An epidemiological study of treatment options for retained deciduous teeth has also shown that the most common treatment option is preservation of the deciduous teeth, followed by orthodontic space closure, implant placement, and other prosthetic procedures [5]. Patients with retained deciduous teeth before orthodontic treatment are considered to have poor long-term prognosis. Originally, the roots of the retained deciduous teeth were shorter than those of permanent teeth; thus, they were often extracted. However, in some cases, long-term retention of deciduous teeth can help establish good individual normal occlusion. Specifically, in low-angle Class II cases that involve the extraction of the retained mandibular second deciduous molars and require large proximal movement of the mandibular first molars, vertical control must be considered during orthodontic treatment, and treatment tends to be difficult [5, 6]. Therefore, a treatment plan that preserves the deciduous teeth for future prosthetic treatments is one of the options. In addition, in cases such as oligodontia, it is desirable to minimise the number of teeth for extraction, except for deciduous teeth that do not remain functional in the long term. It is important to preserve the mandibular second deciduous molars, which have large crowns that can carry the occlusal force if root resorption does not progress in the long term. Therefore, proper diagnosis and prognosis are extremely important.

Root resorption of the permanent teeth is generally reported to be caused by endogenous factors, such as pulpal infection and exogenous factors, such as tooth eruption, ankylosis, orthodontic force, periodontal tissue infection, and tumour pressure [7–10]. In a previous longitudinal study that followed the long-term prognosis of retained deciduous teeth, no correlation or specific pattern was found among age, presence or absence of caries, presence or absence of hypodontia, sex, or root resorption [11]. Subsequent studies have found that retained deciduous teeth with a congenital absence of subsequent permanent teeth are strongly associated with infraocclusion [12]. A recent study reported a correlation between age, infraocclusion, and root resorption in retained deciduous teeth [13].

Thus, the prognostic values and risk factors of root resorption in the mandibular second deciduous molars remain uncertain. The prognosis is vague; thus, dentists must rely on their own assessment to predict the timing of tooth loss. Systemic inflammatory reactions, such as allergies, are known to affect bone and other hard tissues [14]. An association between hypoxia and external root resorption has also been observed [15]. Since there are currently no studies that quantitatively compare the relationship of root resorption to the patient’s systemic history or craniofacial and intraoral morphology, especially in relation to possible host factors, we investigated their relationships in this study. Therefore, we hypothesised that ‘maxillofacial and intraoral morphology and systemic history are factors that cause root resorption of retained mandibular second deciduous molars with congenital absence of subsequent permanent teeth’. This study aimed to clarify the factors related to root resorption of retained mandibular second deciduous molars and predict the prognosis of the teeth.

## Methods

**Fig. 1:**
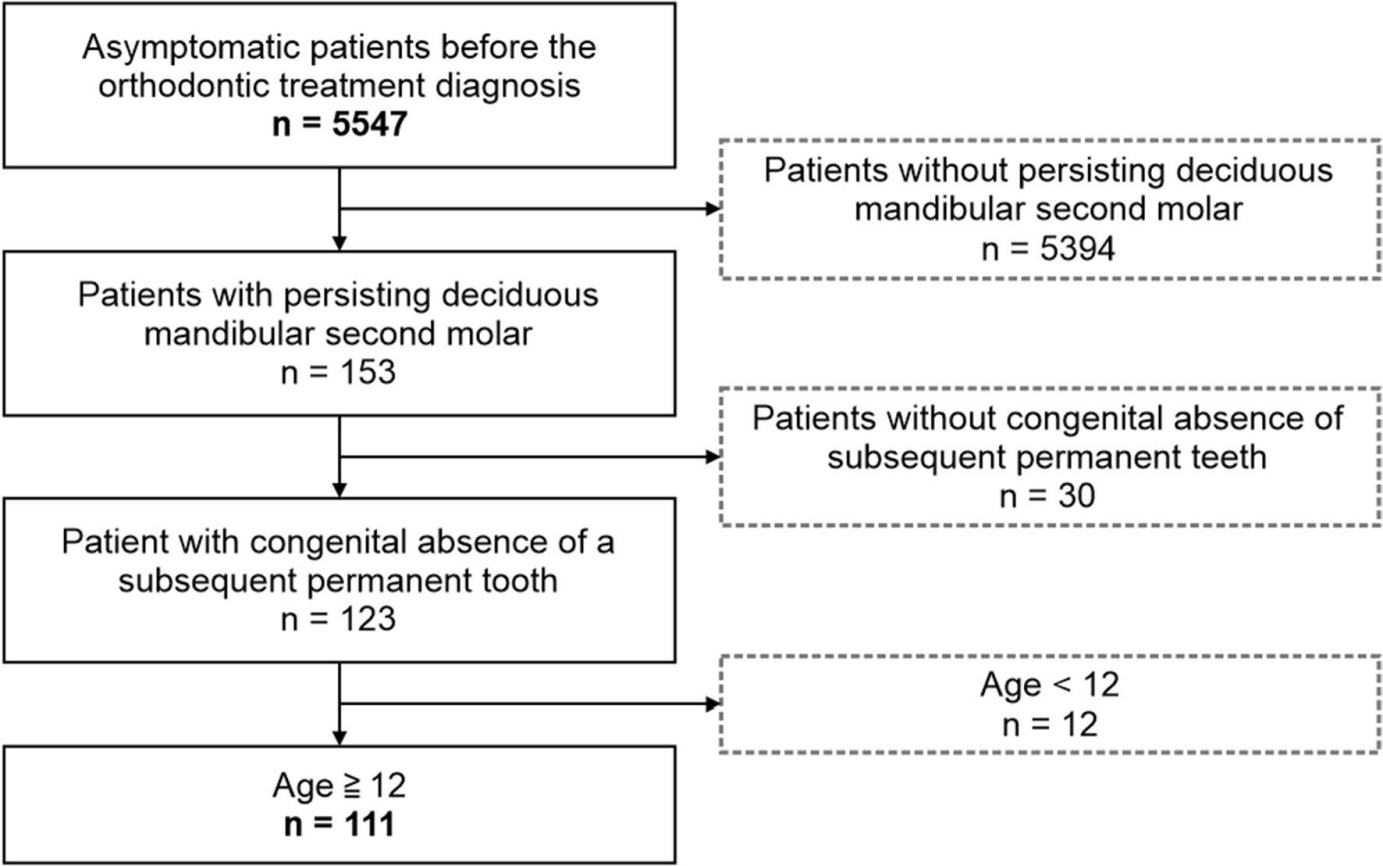
Participant selection flowchart

This cross-sectional study was approved by the Institutional Research Ethics Committee (Permission number: D2022-041) and was conducted in accordance with the principles of the Declaration of Helsinki. Patients and their parents or legal guardians were informed of the possibility that patient records would be used anonymously for teaching and research purposes, and informed consent was obtained. Since the average age at which the completion of the tooth embryo of the mandibular second premolar can be confirmed through radiographs is 3.70 years [16], the mandibular second molar with precession can be identified from this age on panoramic radiographs. We defined late retained deciduous teeth as after the time of eruption of the subsequent permanent second premolor, and decided to study patients aged 12 years and older. Specifically, we screened a cohort of 5547 asymptomatic patients who visited the orthodontics clinic at Tokyo Medical and Dental University Dental Hospital in Tokyo between 2013 and 2022, using a selection flowchart based on the previous studies (Fig. 1).

The inclusion criteria were as follows:

1. congenitally missing mandibular second premolar,
2. retained mandibular second deciduous molars,
3. high-quality pre-treatment radiographs (orthopantomograms and cephalograms),
4. healthy patients between 12 and 32 years old,
5. Japanese (Mongoloid).

The exclusion criteria were as follows:

1. severe skeletal dysmorphism or malformation,
2. a history of orthodontic or orthognathic treatment,
3. cranial anomalies or craniofacial syndromes.

Finally, 111 patients (i.e. 156 teeth) were selected for the analyses.

### Variables for measurement

**Fig. 2:**
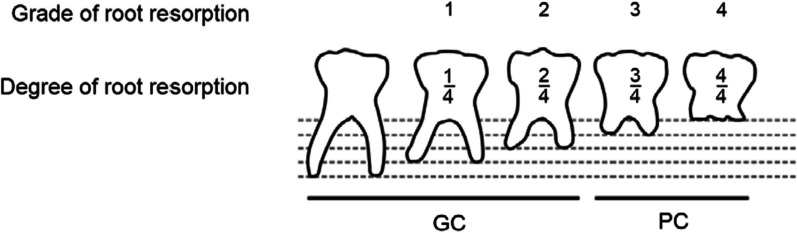
Definition of groups for the retained mandibular second deciduous molar. Group indicators are set according to the amount of root resorption. Teeth with over 3/4 root resorption were classified into the poor condition (PC) group, while those with less than 2/4 root resorption were classified into the good condition (GC) group

**Fig. 3:**
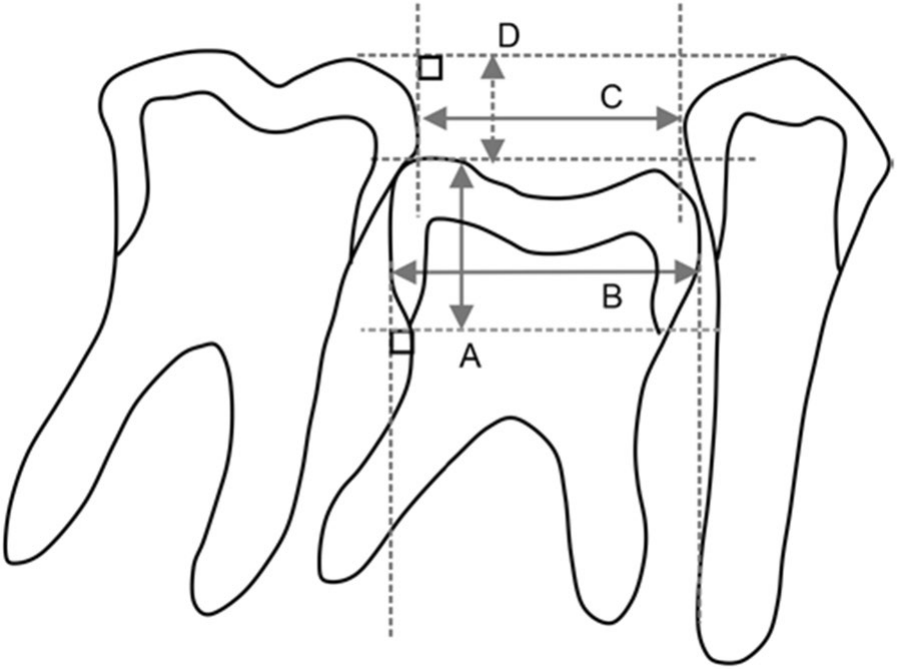
Definitions of parameters for the subject teeth on panoramic radiographs. A line from the apex of the second deciduous molar to the cemento-enamel junction (**A**) was used as the *y*-axis, which was defined as crown height, and the *x*-axis was perpendicular to it. The distance from the mesial to the distal surface of the second deciduous molar on the *x*-axis was defined as crown width (**B**). Furthermore, the distance from the distal surface of the first premolar to the mesial surface of the first molar was defined as the interdental space (**C**), and the distance from the line connecting the apex of the first premolar and the first molar perpendicular to it to the apex of the mandibular second deciduous molar was defined as the distance from the occlusal plane (**D**). A; crown height, B; crown mesiodistal width, C; adjacent interdental space, and D; distance from the occlusal plane

Lateral cephalometric and panoramic radiographs, upper and lower dental models, and orthodontic treatment questionnaires were used as references. All cephalometric and panoramic radiographs were captured using the same independent X-ray apparatus. First, the subject teeth were divided into two groups based on their root resorption levels from panoramic radiographs using Bjerklin and Bennett's classification [17], as shown in Fig. 2: good condition (GC) group (38 teeth in males and 45 teeth in females) with one and two grades of root resorption, which had less root resorption, and the poor condition (PC) group (22 teeth in males and 51 teeth in females) with three and four grades of root resorption, which had more root resorption. The crown height, crown mesiodistal width, adjacent interdental space, and distance from the occlusal plane were measured from panoramic radiographs using callipers [12, 18, 19] (Fig. 3). Distance from the occlusal plane was defined as the number of teeth below the occlusal plane [20] and measured in millimetres using the following method. First, a line was drawn between the cusp images to define the occlusal table of the infraoccluded teeth. A line parallel to the connecting occlusal planes of the two adjacent teeth was drawn on the infraoccluded teeth. For teeth that were not infraoccluded, the distance from the occlusal plane was set to 0 mm. If the adjacent teeth were angulated, a line was drawn between the tips of the highest cusps of the adjacent teeth. The vertical dimension between the line of the occlusal planes of the adjacent teeth and the median plane of the occlusal teeth was measured using a calliper with an accuracy of 0.1 mm. Mesiodistal dimensions were measured on dental casts using callipers (Mitutoyo, Kanagawa, Japan). Each measurement was performed twice on each tooth, and if the discrepancy was more than 0.4 mm, the measurement was repeated. The mesiodistal distance was defined as the greatest distance between the contact points of the tooth crown. The SNA/SNB/ANB/Frankfort-mandibular plane angle (FMA) and U1 to FH/L1 to mandibular/Frankfort-mandibular incisor angle (FMIA) values, which are indicators of craniofacial position and relationship, such as maxillary, mandibular, and maxillomandibular skeletal characteristics, and anterior teeth angle, respectively, were measured by cephalometric analysis. All cephalometric radiographs were obtained according to the internationally popular settings. When obtaining cephalometric radiographs, the patient’s head was fixed with ear rods, and the Frankfort horizontal plane was set parallel to the floor. The distances between the light source and the subject and between the subject and the film were always fixed [21, 22]. A model analysis was performed using a dental cast to measure the amount of overbite, overjet, and discrepancy between the upper and lower jaws. Systemic and dental history were obtained from the pre-treatment questionnaire, such as age, sex, number and laterality of congenitally missing teeth, allergy, presence of mouth breathing habits, history of dental caries or endodontic treatment, and Epworth Sleepiness Score (a self-administered questionnaire which provides a measurement of the participant’s general level of daytime sleepiness which is associated with obstructive sleep apnoea [23]). Age was calculated in days, divided by 365.25, and computed up to two decimals.

### Statistical analyses

All measurements of the lateral cephalometric radiographs were traced and performed by a single investigator (KI) using Winceph ver.9.0 software (Rise Corp., Tokyo, Japan). One month later, 30 radiographs and dental casts were randomly chosen, and the measurements were repeated by the same investigator to evaluate the intra-examiner reliability. There were no significant differences between the first and second measurements. All parameters were randomly re-measured at 2-month intervals. The intraclass correlation coefficient (ICC) and Dahlberg’s formula [24] were used to evaluate measurement error. On average, the method error was 0.03 mm for distance from the occlusal plane, 0.21 mm for adjacent interdental space, 0.41 degrees for FMA, 0.36 degrees for FMIA, and 0.34 mm for overbite. The results of the random and systematic error assessments showed excellent intra-examiner agreement; the ICC values for all measurements were above 0.90, indicating low measurement error. Statistical analyses were performed using SPSS Statistics for Windows, version 15.0 (SPSS Inc., Chicago, Ill., USA) at a significance level of 5%. Univariate logistic regression models were used, wherein the changing amount of root resorption was regressed on the differences in demographic, clinical, cephalometric, and dental cast parameters. Subsequently, multivariate logistic regression models were developed to estimate the risk of root resorption associated with potential predictors, including craniofacial characteristics and patient history. The inclusion of variables in the models was based on the existing knowledge of the relevance of root resorption in patients with congenital mandibular second premolars. The patient characteristics of the PC and GC groups were compared. Sample size calculation was performed based on logistic regression analysis. With an alpha error of 0.05 and a power of 0.8, a sample size of 104 would be required to detect a statistically significant odds ratio (OR) of 1.75. Additionally, it necessitated at least 10 outcomes for each included independent variable, a criterion that was also met [25]. The model’s predictive performance was evaluated by assessing its discriminatory ability. Discrimination was measured using the area under the curve of the receiver operating characteristic (AUC) [26].

## Results

### Baseline characteristics

Table 1 shows the baseline characteristics and distributions. The participants are aged between 12 and 32 years, with a mean age of 18.7 ± 5.1 years [< 20 years, 106 (67.7%); ≥ 20 years, 50 (32.1%)]. The incidence of congenitally missing mandibular second premolars with retained mandibular second deciduous molars at our clinic was 2.0% (123/5547). The number of patients in each group is presented in Table 2. In total, 111 patients (156 teeth) were included in the analysis. Eighty-three teeth (53.2%, 83/156) exhibited GC, whereas 73 teeth exhibited PC (46.8%, 73/156). The mean and standard deviation of the analysed values from the cephalometric analysis, model analysis, and measurements of the panoramic radiographs are summarised in Table 3.

**Table 1:** Patients’ characteristics

Factors	Total (n = 156)
Demographic	
Age (years, mean ± SD)	18.7 ± 5.1
< 20 (%)	106 (67.9)
≧ 20 (%)	50 (32.1)
Sex	
Male (%)	60 (38.5)
Female (%)	96 (61.5)
Dental	
#75 (%)	77 (49.3)
#85 (%)	79 (50.7)
Clinical	
Hypodontia (%)	110 (70.5)
Oligodontia (%)	46 (29.5)
Allergy	
Yes (%)	34 (21.8)
No (%)	122 (78.2)
Mouth breathing	
Yes (%)	32 (20.5)
No (%)	124 (79.5)
ESS (point, mean ± SD)	6.4 ± 3.7
< 11 (%)	126 (80.8)
≧ 11 (%)	30 (19.2)
Laterality	
Unilateral	66 (42.0)
Bilateral	90 (58.0)
Caries treatment	
Yes (%)	39 (25.0)
No (%)	117 (75.0)
Endodontic treatment	
Yes (%)	6 (3.8)
No (%)	150 (96.2)

ESS, Epworth sleepiness score and SD, standard deviation

**Table 2:** Number of teeth per group

Demographic	GC	PC
Age		
< 20	63	43
≧ 20	20	30
Sex		
Male	38	22
Female	45	51
Dental		
#75	39	38
#85	44	35
Clinical		
Hypodontia	56	54
Oligodontia	27	19
Allergy		
Yes	20	14
No	63	59
Mouth breathing		
Yes	16	15
No	68	57
ESS		
< 11	70	56
≧ 11	13	17
Laterality		
Unilateral	33	33
Bilateral	50	40
Caries treatment		
Yes	9	26
No	74	47
Endodontic treatment		
Yes	3	2
No	80	71

ESS, Epworth sleepiness score; PC, poor condition; and GC, good condition

**Table 3:** Cephalometric measurements

*Cephalometric*	
SNA (degree)	79.8 ± 3.7
SNB (degree)	77.3 ± 3.9
ANB (degree)	2.4 ± 3.2
FMA (degree)	28.3 ± 7.0
U1 to FH (degree)	111.5 ± 9.8
L-1 to mandibular plane (degree)	88.4 ± 12.8
FMIA (degree)	62.2 ± 10.0
*Model analysis*	
Overjet (mm)	2.9 ± 3.3
Overbite (mm)	2.9 ± 2.5
Upper arch length discrepancy (mm)	2.1 ± 8.3
Lower arch length discrepancy (mm)	1.6 ± 7.2
*Panoramic*	
Crown height (mm)	6.4 ± 0.8
Crown mesiodistal width (mm)	12.9 ± 1.3
Adjacent interdental space (mm)	8.9 ± 2.2
Distance from occlusal plane (mm)	2.2 ± 2.7

Values are depicted in mean ± SD

FMA, Frankfort-mandibular plane angle; FMIA, Frankfort-mandibular incisor angle; and SD, standard deviation

### Univariate logistic regression analysis

Table 4 shows the ORs for predictive factors for increased root resorption in the univariate logistic regression analysis. The patient characteristics of the PC and GC groups were compared. FMA was significantly associated with an increase in root resorption [OR: 0.865, 95% confidence interval (CI) 0.769–0.974]. This indicates that a smaller FMA is associated with a higher risk of increasing root resorption. Overbite was also significantly associated with an increase in root resorption (OR: 1.321, 95% CI 1.053–1.658), suggesting that a larger overbite is associated with a higher risk of increasing root resorption. Moreover, an increase in adjacent interdental space (OR: 1.439, 95% CI 1.049–1.973) and distance from the occlusal plane (OR: 0.785, 95% CI 0.617–1.000) were found to be significantly associated with an increase in root resorption. Additionally, caries treatment was significantly associated with an increase in root resorption (OR: 7.651, 95% CI 1.922–30.457).

**Table 4:** Univariate logistic regression analysis in the GC and PC groups

Exposures	Univariate logistic regression		
Demographic	OR	95% CI	*p*
Age			
< 20	0.99	0.90–1.08	0.82
≧ 20			
Sex			
Male (%)	2.10	0.72–6.15	0.17
Female (%)			
Dental			
#75	0.88	0.32–2.40	0.80
#85			
Clinical			
Hypodontia	1.55	0.41–5.87	0.51
Oligodontia			
Allergy			
Yes	0.82	0.21–3.26	0.78
No			
Mouth breathing			
Yes	0.20	0.053–1.29	0.87
No			
ESS (points)	1.60	0.37–6.92	0.52
Laterality			
Unilateral	0.78	0.26–2.38	0.67
Bilateral			
Caries treatment			
Yes	7.65	1.92–30.45	< 0.01*
No			
Endodontic treatment			
Yes	1.45	0.10–20.66	0.78
No			
Cephalometric			
SNA (degree)	1.21	0.94–1.56	0.13
SNB (degree)	0.93	0.70–1.23	0.61
ANB (degree)	1.07	0.81–1.42	0.61
FMA (degree)	0.86	0.76–0.97	0.01*
U1 to FH (degree)	1.02	0.93–1.10	0.60
L-1 to mandibular plane (degree)	0.38	0.90–1.04	0.96
FMIA (degree)	0.93	0.84–1.03	0.17
Model analysis			
Overjet (mm)	0.95	0.72–1.26	0.75
Overbite (mm)	1.32	1.05–1.65	0.01*
Upper arch length discrepancy (mm)	1.06	0.97–1.15	0.17
Lower arch length discrepancy (mm)	0.94	0.85–1.04	0.25
Panoramic			
Crown height (mm)	0.46	0.21–1.00	0.05
Crown mesiodistal width (mm)	1.30	0.83–2.03	0.24
Adjacent interdental space (mm)	1.43	1.04–1.97	0.02*
Distance from occlusal plane (mm)	0.78	0.61–1.00	0.05

ESS, Epworth sleepiness score; FMA, Frankfort-mandibular plane angle; FMIA, Frankfort-mandibular incisor angle; CI, confidence interval; OR, odds ratio; SD, standard deviation; GC, good condition group; and PC, poor condition group

*p < 0.05

### Multivariate logistic regression analysis

Clinical, demographic, cephalometric, panoramic, and dental cast parameters were included in the multivariable logistic regression analysis. Finally, a model predicting increased root resorption based on the number of factors associated with increased root resorption that was included in the multivariable logistic regression model was developed (Table 5). The multivariate ORs for distance from the occlusal plane, adjacent interdental space, FMA, FMIA, overbite, and caries treatment were statistically significant; the other parameters showed no significant ORs. In particular, a decrease in the values of distance from the occlusal plane, FMA, and FMIA, along with an increase in the values of overbite, adjacent interdental space, and a history of dental caries treatment, was found to be significantly associated with increased root resorption in retained mandibular second deciduous molars. In addition, we evaluated the usefulness of this model. A receiver operating characteristic (ROC) curve [26] was generated, and an AUC value of 0.84 was obtained, indicating that this model has high predictability (Table 6).

**Table 5:** Multivariable logistic regression analysis in the GC and PC groups

Exposures	OR	95% CI	*p*
Age	0.98	0.92–1.05	0.67
Sex	1.52	0.61–3.73	0.36
Distance from occlusal plane (mm)	0.80	0.66–0.97	0.02*
Adjacent interdental space (mm)	1.46	1.15–1.85	< 0.01*
FMA (degree)	0.87	0.80–0.95	< 0.01*
FMIA (degree)	0.93	0.88–0.98	< 0.01*
Overbite (mm)	1.38	1.13–1.67	< 0.01*
Caries treatment	7.05	2.93–20.19	< 0.01*

FMA, Frankfort-mandibular plane angle; FMIA, Frankfort-mandibular incisor angle; CI, confidence interval; OR, odds ratio; SD, standard deviation; GC, good condition group; and PC, poor condition group

*p < 0.05

**Table 6:** Area under the receiver operating characteristic curve outcome

AUC	95% CI
0.84 ± 0.32	0.77–0.90

CI, confidence interval and AUC, area under the receiver operating characteristic curve

## Discussion

Root resorption of deciduous teeth is a clinically important physiological process in the eruption sequence for the acquisition of normal permanent dentition; however, its importance is often overlooked because of the presence of permanent teeth. This is the first cross-sectional study to investigate the factors that contribute to root resorption in retained mandibular second deciduous teeth with congenital absence of subsequent permanent teeth based on the patient's systemic history, craniofacial and intraoral morphology, and possible host factors.

More accurate measurements could have been obtained if standardised radiographs, rather than panoramic radiographs, were taken periodically. However, routine radiographs are not indicated for the examination of orthodontic patients and may not be ethically justified due to radiation exposure. Therefore, following the previous studies that reported less statistical error in certain horizontal measurements [27], we opted to use panoramic radiographs for this study. Additionally, it has been reported that there is minimal change in magnification, especially in the mandibular first molar regions [28, 29].

The incidence of the congenital absence of the second premolars was 2.0% in our study, which is consistent with a meta-analysis by Polder et al. [29, 30]. The sex differences were 38.5% for males and 61.5% for females, with approximately 20% more females. This result is consistent with those of the previous studies, although it may be due to the selection criteria used for the orthodontic patient population [11]. The present study suggests that maxillofacial and intraoral morphologies may play a role in the root resorption of retained mandibular second deciduous teeth with the congenital absence of subsequent permanent teeth. Smaller distance from the occlusal plane, smaller FMA, smaller FMIA, larger adjacent interdental space, larger overbite, and a history of caries treatment were associated with an increase in the amount of root resorption. An ROC curve was developed to identify the model’s predictive ability, and the AUC value of this model was 0.84. This curve and the corresponding AUC show that distance from the occlusal plane, FMA, FMIA, adjacent interdental space, overbite, and caries treatment have the predictive ability to discriminate the risk of root resorption in retained mandibular second deciduous teeth with congenital absence of subsequent permanent teeth.

In this study, factors influencing root resorption in retained deciduous second molars included a large overbite and small FMA and FMIA, contributing to an increase in root resorption. Patients with hypodontia often exhibit a clockwise rotation of the mandible due to vertical facial development deficiency, arising from reduced cranial base length and angle, along with diminished alveolar bone length as a growth modification and posterior occlusal support [4]. Additionally, patients with maxillofacial morphology characterised by a small FMA and deep overbite generally tend to exert strong occlusal forces [31]. Excessive occlusal forces can lead to traumatic occlusion, wherein functional or parafunctional forces damage the periodontal ligament (PDL), surpassing the tooth’s ability to repair itself [32]. In addition, patients with labially inclined mandibular anterior teeth may experience inadequate occlusal contact with these teeth, resulting in increased occlusal forces in the molar region [33].

The present study suggests that a smaller distance from the occlusal plane and larger adjacent interdental spaces is associated with increased root resorption. A large interdental space denotes that the discrepancy is likely to be small, and the distance from the occlusal plane is also likely to be small (i.e. the occlusal plane of the deciduous tooth is close to the occlusal plane of the dentition). Both intraoral environments allow the occlusal forces to be more likely exerted on the deciduous tooth. In the present study, data regarding missing teeth next to the deciduous second molars were excluded; there were no isolated deciduous second molars. Increased occlusal load induces an inflammatory microenvironment responsible for root apex tissue resorption [34]. Although the mechanism of physiological root resorption in deciduous teeth is not yet clear, it is recognised as a complex physiological process regulated by multiple cytokines and transcription factors in the inflammatory microenvironment through various signalling pathways. These pathways involve hard tissue resorption, such as dentin and cementum, as well as soft tissue resorption, such as pulp and PDL [31, 34–36]. Some of these inflammatory stimuli, including mechanical stress on deciduous teeth during masticatory movements such as chewing, may also be a possible cause.

The previous studies demonstrated root resorption in animal models when bite force was applied, leading to periodontal tissue dysfunction owing to occlusal insufficiency [37, 38]. During facial growth and development, mechanical stress on deciduous teeth increases gradually. Excessive mechanical stress may upregulate receptor activators of nuclear factor kappa-Β ligand expression in PDL cells, promoting osteoclast formation and root resorption [36, 37]. To minimise root resorption, it is necessary to apply appropriate occlusal forces tolerable by the PDL tissues of deciduous teeth. The appropriate occlusal force that does not significantly increase apoptosis is known to be 0–180 kPa, tolerated by the PDL of permanent teeth, but not more than approximately 0–135 kPa for deciduous teeth [39]. Appropriate occlusal adjustment or force loading of the deciduous teeth for long-term retention may also reduce root resorption.

Our findings differ from those in a study by Hvaring et al. [13], reporting that retained deciduous teeth with congenitally absent succeeding teeth were associated with increased root resorption and infraocclusion. To explain this paradox, we need to consider it in relation to the mechanism of disuse atrophy of the alveolar bone and tooth root. Bone loss occurs in the alveolus when functional occlusal pressure is reduced. Adjacent teeth with reduced bone loss tend to be more inclined [40] and have smaller interdental space [20], potentially related to a lower occlusal surface. The second deciduous molars, carrying the occlusal load during the deciduous period, may become infraoccluded with the eruption and growth of permanent teeth, no longer bearing occlusal force load. If the molars with resorption were in infraocclusion, the received occlusal forces would be indirect from adjacent teeth. As reported, adjacent teeth of infraoccluded teeth are progressively inclined [40] and subjected to occlusal forces in various directions depending on occlusal surface morphology. In such cases, infraoccluded teeth are likely subjected to mesial and distal occlusal forces from adjacent teeth. Thus, it is assumed that the amount of root resorption of the retained mandibular second deciduous molar increases due to the received occlusal load in both the previous studies and the present study.

History of caries treatment is associated with changes in root resorption. It has been reported that caries treatment of molars increases occlusal contact due to composite resin and other occlusal surface morphologies, as well as the burden on the teeth compared to untreated conditions, such as pulp irritation due to caries. Although different from the report of Rune et al. [11], their study focused only on the presence or absence of ‘caries’ and did not elucidate the treatment history. A previous study indicated the impact of genetic aetiology [41] and that changes in proteins [cluster of differentiation 14 and 3] in the remaining dental pulp of deciduous molars in animal models which have been extracted permanent teeth play an important role in increased root resorption [42]. Therefore, future studies, such as genetic data analysis and basic physiological research, are warranted to clarify the details of root resorption in retained mandibular second deciduous teeth with the congenital absence of subsequent permanent teeth. With 228 partially edentulous patients accounting for up to 180 different patterns of missing teeth [38], the patterns of missing teeth among these patients differed extensively. In cases where several consecutive teeth are missing, or there are concomitant abnormalities in the crown morphology, such as dwarf teeth, it is often difficult to achieve space closure by tooth movement alone. Therefore, it is necessary to develop a treatment plan that combines multiple options, such as prosthetics, autotransplantation, and preservation of deciduous teeth. Preservation of deciduous teeth is also a non-invasive option that is the least expensive and may leave space closure with implants or orthodontic treatment as a future option [39].

### Limitations

The random selection of participants and generalisability of our study are limited in the following respects: This study was conducted in Japanese patients who were assumed to have started orthodontic treatment. Therefore, these results cannot be extrapolated to other ethnic populations. Furthermore, a male-to-female ratio was observed, and the target age group is rather large. Additionally, as this was a cross-sectional and exploratory study, research with longitudinal findings and basic experimental studies are still warranted in future.

## Conclusions

Although there are some limitations, the findings of this study indicate that FMA, FMIA, overbite, distance from the occlusal plane, adjacent interdental space, and a history of dental caries treatment are factors contributing to the root resorption of the retained mandibular second deciduous tooth with congenital absence of the subsequent permanent tooth. Therefore, when planning orthodontic treatment for patients with congenitally absent mandibular second premolar, orthodontists should consider the long-term preservation of the retained mandibular second deciduous molar based on the individual’s defect and maxillofacial morphology.

## Data Availability

The datasets underlying this article are available upon reasonable request to the corresponding author.

## Abbreviations

GC: Good condition
PC: Poor condition
ROC: Receiver operating characteristic
AUC: Area under the curve
PDL: Periodontal ligament
FMA: Frankfort-mandibular plane angle
FMIA: Frankfort-mandibular incisor angle
ICC: Correlation coefficient
CI: Confidence interval
OR: Odds ratio
